# Honor to Dr. Jenkins

**Published:** 1903-12

**Authors:** E. A. Bogue


### Domestic Correspondence.

### HONOR TO DR. JENKINS.

To the Editor:

Sir,—There may be simultaneous invention or discovery in various parts of the world, but the man that puts time, money, brains, and influence into the work of *introducing* a good thing, and making it known, is the man who should receive the honor.

If, in addition, he puts himself earnestly, honestly, and intelligently to the improvement of the product, he is all the more a benefactor.

This is apropos of the modest letter from Dr. N. S. Jenkins on page 560 of the July International.

I happen to have seen some of the work done by Dr. Jenkins in his efforts to perfect the beautiful branch of our art upon which he has been engaged so many years, and I am fully convinced that he will presently furnish dentists with an enamel sufficiently low fusing to be worked with ease, but sufficiently near the high-fusing bodies to be durable in the mouth, and to be reliable in the matter of keeping its color under the necessary heat.

Furthermore, I believe that Dr. Jenkins's discoveries in this direction are to result in a revival of the use of that most artistic and beautiful substitute for lost teeth, continuous gum work, which can be used for partial plates as well as complete ones.

E. A. Bogue.

